# Genetic mapping identified three hotspot genomic regions and candidate genes controlling heat tolerance-related traits in groundnut

**DOI:** 10.3389/fpls.2023.1182867

**Published:** 2023-05-23

**Authors:** Vinay Sharma, Sunil S. Gangurde, Spurthi N. Nayak, Anjan S. Gowda, B.S. Sukanth, Supriya S. Mahadevaiah, Surendra S. Manohar, Rakeshkumar S. Choudhary, T. Anitha, Sachin S. Malavalli, S.N. Srikanth, Prasad Bajaj, Shailendra Sharma, Rajeev K. Varshney, Putta Latha, Pasupuleti Janila, Ramesh S. Bhat, Manish K. Pandey

**Affiliations:** ^1^Center of Excellence in Genomics & Systems Biology (CEGSB), International Crops Research Institute for the Semi-Arid Tropics (ICRISAT), Patancheru, Hyderabad, India; ^2^Department of Genetics and Plant Breeding, Chaudhary Charan Singh University (CCSU), Meerut, India; ^3^Department of Biotechnology, University of Agricultural Sciences, Dharwad, India; ^4^Regional Agricultural Research Station, Acharya N G Ranga Agricultural University (ANGRAU), Tirupati, India

**Keywords:** heat stress — tolerance — molecular markers — QTL, genotyping by sequencing, candidate gene, peanut, genomic regions

## Abstract

Groundnut productivity and quality have been impeded by rising temperatures in semi-arid environments. Hence, understanding the effects and molecular mechanisms of heat stress tolerance will aid in tackling yield losses. In this context, a recombinant inbred line (RIL) population was developed and phenotyped for eight seasons at three locations for agronomic, phenological, and physiological traits under heat stress. A genetic map was constructed using genotyping-by-sequencing with 478 single-nucleotide polymorphism (SNP) loci spanning a map distance of 1,961.39 cM. Quantitative trait locus (QTL) analysis using phenotypic and genotypic data identified 45 major main-effect QTLs for 21 traits. Intriguingly, three QTL clusters (Cluster-1-Ah03, Cluster-2-Ah12, and Cluster-3-Ah20) harbor more than half of the major QTLs (30/45, 66.6%) for various heat tolerant traits, explaining 10.4%–38.6%, 10.6%–44.6%, and 10.1%–49.5% of phenotypic variance, respectively. Furthermore, important candidate genes encoding *DHHC-type zinc finger family protein* (*arahy.J0Y6Y5*), *peptide transporter 1* (*arahy.8ZMT0C*), *pentatricopeptide repeat-containing protein* (*arahy.4A4JE9*), *Ulp1 protease family* (*arahy.X568GS*), *Kelch repeat F-box protein* (*arahy.I7X4PC*), *FRIGIDA-like protein* (*arahy.0C3V8Z*), and post*-illumination chlorophyll fluorescence increase* (*arahy.92ZGJC*) were the underlying three QTL clusters. The putative functions of these genes suggested their involvement in seed development, regulating plant architecture, yield, genesis and growth of plants, flowering time regulation, and photosynthesis. Our results could provide a platform for further fine mapping, gene discovery, and developing markers for genomics-assisted breeding to develop heat-tolerant groundnut varieties.

## 1 Introduction

Groundnut or peanut (*Arachis hypogaea* L.) is a major oilseed, food, and fodder crop, especially for smallholder farmers in Asia and Africa’s semi-arid tropics (SAT) regions. In recent years, the global temperature has risen, which is leading to drought and high-temperature stress in SAT regions (Puppala et al., 2023). Approximately 90% of the total groundnut is cultivated in the SAT regions of the world. Temperatures in the majority of SAT regions exceed the critical threshold during reproductive and pod-filling stages, which results in significant yield losses in these regions (Hamidou et al., 2013; Akbar et al., 2017). Climate change prediction studies in these semi-arid regions revealed a steady decline in production, which places food security in these regions at risk (Howden et al., 2007). Crop yields are expected to decline by 15%–35% in Asia and Africa and by 25%–35% in the Mid-East as a result of a 3°C–4°C increase in temperature (Ortiz et al., 2008). Existing approaches for mitigating high-temperature stress through technological and management systems are insufficient to maintain yields (Driedonks et al., 2016). Heat stress restricts sucrose transport, resulting in a sucrose shortage in the reproductive organs, and impairs the developmental and functional processes of flowers, pods, and seed filling (Kaushal et al., 2013). Groundnut photosynthesis and vegetative growth are well adapted to high temperatures, and the optimal mean daily temperature for such processes is between 30°C and 35°C. Conversely, groundnut reproductive processes are highly sensitive to elevated air temperature, like other legumes such as common beans and cowpea and also cereals such as rice (Craufurd et al., 2003). During flower development, microsporogenesis (3–6 days prior to the emergence of flowers) and anthesis are particularly more sensitive to high temperatures in groundnut (Prasad et al., 2001). As pod and kernel development expand to the underground region, the pod development is influenced by the soil temperature. For proper flower development, the genotype should have tolerance for high air temperature. The optimal temperature for pod and kernel development is approximately 23°C (Cox, 1979), which is substantially lower than the temperature required for vegetative growth and development. Elevated temperature in soil (>38°C) decreases the accumulation of dry matter, yield, the percent of pegs developing pods, and seed mass per seed (Vara Prasad et al., 2000).

The development of heat-tolerant varieties would be a sustainable approach to overcoming yield losses due to heat stress. Heat-tolerant cultivars can perform normal physiological processes and produce a stable yield at elevated temperatures (Wahid and Close, 2007). Heat tolerance is described as the ability of plants to sustain critical functions while the tissues become warm (Hall, 1992; Mahan et al., 1995). It is important to identify heat-tolerant genotypes and surrogate traits to successfully breed heat-tolerant cultivars. Among several traits, pod yield is the most promising selection measure under stress conditions. Genotypes that exhibit the least yield loss in both stress and non-stress regimes are found to be potential sources. However, in groundnut, the pods are underground, making it difficult to estimate production performance until harvesting. Thus, it is important to identify surrogate traits that improve yield performance in the initial stages of stress. This will allow early selection and provide a useful source for tolerant genotypes/population development. There have been reports of genotypes with genetic variations for dry matter partitioning to pods and kernels, fruit setting, thermo-stability of the cellular membrane, and chlorophyll fluorescence in groundnut. Identification and utilizing genetic diversity found in landraces, cultivated varieties, advanced breeding lines, and crop wild relatives are essential to improving heat-stress tolerance (Gautam et al., 2022). The complexity of the polygenic nature of the trait, its low heritability, and the extensive genotype × environment interactions limit the development of high-yielding cultivars for locations with heat stress (Blum, 1998). Under such situations, quantitative trait locus (QTL) mapping and their introgression can aid in improving complex traits like yield and surrogate traits. Genetic mapping dissects the genomic regions associated with yield and surrogate traits in targeted environments, fine map the genomic regions, and identify molecular markers strongly associated with the trait of interest for molecular breeding (Soriano et al., 2017).

Utilization of genomic resources and modern breeding strategies, such as marker-assisted selection (MAS) (Varshney et al., 2014; Shasidhar et al., 2020), genomic selection (Pandey et al., 2020a), and rapid generation advancements (Parmar et al., 2021), has streamlined the groundnut breeding program. Furthermore, the availability of reference genomes for cultivated tetraploid groundnut (Bertioli et al., 2019; Chen et al., 2019; Zhuang et al., 2019), along with advancements in next-generation sequencing, such as genotyping-by-sequencing (Wang et al., 2021; Zhou et al., 2021), and high-density Axiom_*Arachis*’ single-nucleotide polymorphism (SNP) arrays (Pandey et al., 2017), significantly lowered the cost of sequencing enabling high-throughput genotyping for high-resolution genetic mapping in groundnut (Pandey et al., 2020a). Several studies have reported QTLs for yield and component traits in various crops like rice (Ye et al., 2012), tomato (Xu et al., 2017), and chickpea (Paul et al., 2018) under heat stress; however, there is no study that reported QTLs/genes associated with surrogate traits of heat stress tolerance in groundnut. A total of 625 diverse groundnut genotypes were screened for high-temperature tolerance under irrigation during the hottest months (February to May) (Ntare et al., 2001). A large variation was observed among the 625 genotypes for pod yield and physiological traits. The crop growth rate was a decisive factor influencing pod yield. Eight genotypes were identified, including two released cultivars (55-437 and 796) in Sahel West Africa with higher pod yield and partitioning coefficient (Ntare et al., 2001). Therefore, in order to identify the QTL associated with surrogate traits of heat tolerance in groundnut, here, we developed a recombinant inbred line (RIL) population by crossing JL 24 (recipient parent) and 55-437 (donor parent) cultivars. The RIL population, along with both parents, was phenotyped in late post-rainy and rainy seasons at three different locations. Genotyping data were generated using genotyping-by-sequencing (GBS) to construct a genetic map and identification of QTLs associated with agronomic, phenological, and physiological traits under heat stress.

## 2 Material and methods

### 2.1 Selection of parents and development of RIL population

A RIL population (JL 24 × 55-437) comprising 248 lines was developed and advanced by single seed descent method up to F_7_:F_8_. The parent cultivar 55-437 (SAMNUT-14), a popular African cultivar developed by I.A.R. Samaru, Zaria, is a medium-early maturing variety (90 to 110 days), is tolerant to drought and high-temperature, and also showed resistance to *Aspergillus flavus* infection. It has the potential to yield up to 20–28 quintals per hectare with approximately 50% of oil content (Craufurd et al., 2003; Ncube Kanyika et al., 2015). This parent was selected and used as the donor parent based on its tolerance to high air temperature at both stages of development, i.e., flowering and seed set (Craufurd et al., 2003). The first flowers of this variety appear approximately 30 to 35 days after sowing (DAS), and the peak flowering period occurs between 45 and 55 DAS. The flowering time is approximately 25 to 30 days. While recipient parent JL 24 with ecotype Spanish bunch is a selection made from genotype EC 94943 (Motagi et al., 2014), it is a popular short-duration cultivar (99–104 days) (Patil et al., 1980). Typically, the JL 24 variety has a flowering duration of approximately 20 to 25 days; the first flowers appear at approximately 30 to 35 DAS, and the peak flowering occurs between 45 and 50 DAS.

### 2.2 Multi-environment evaluation and phenotyping

**Figure 1:**
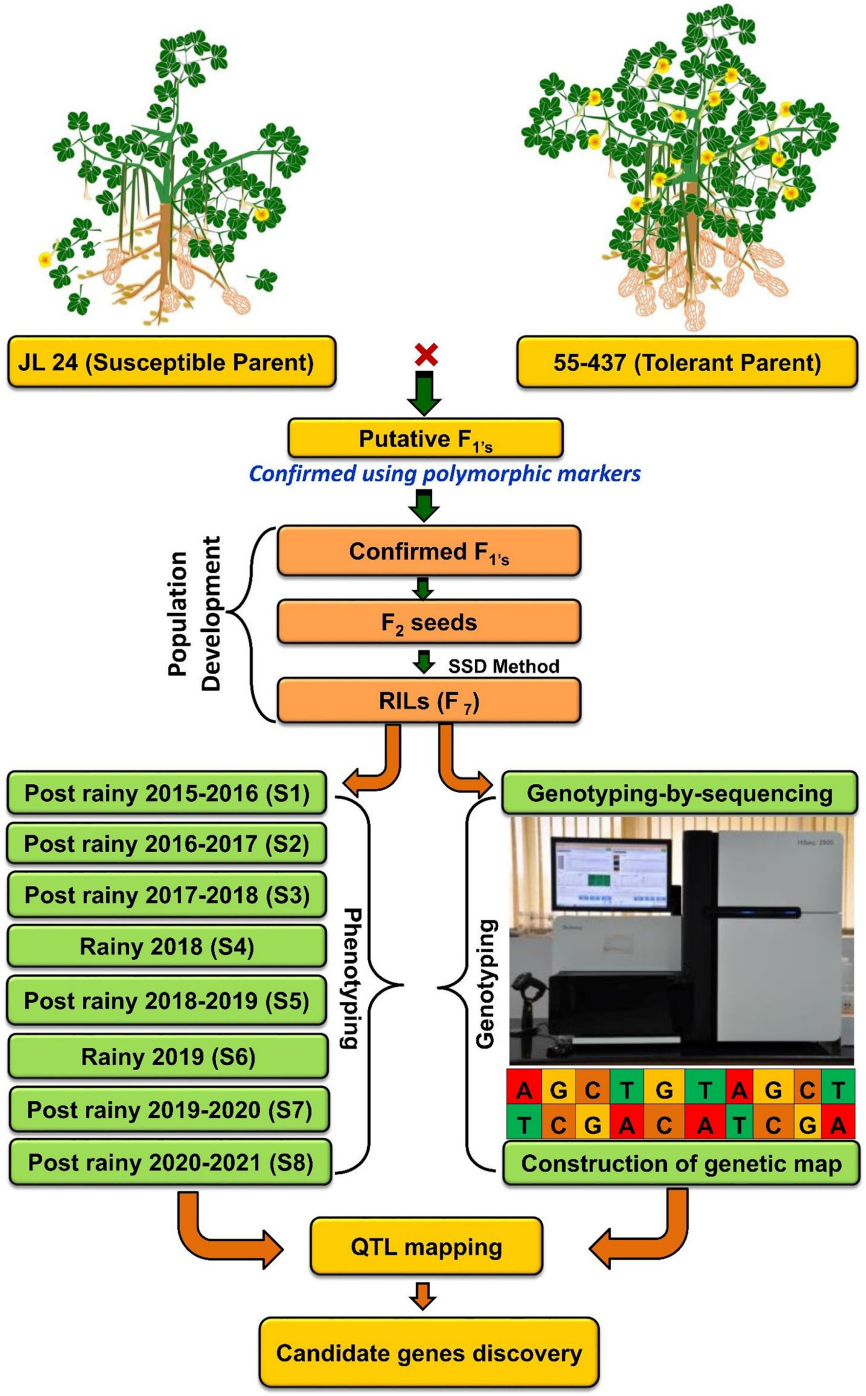
Flowchart of RIL population development, genotyping, and phenotyping for agronomic, phenological, and physiological traits for eight seasons at three locations under heat stress. RIL, recombinant inbred line.

**Figure 2:**
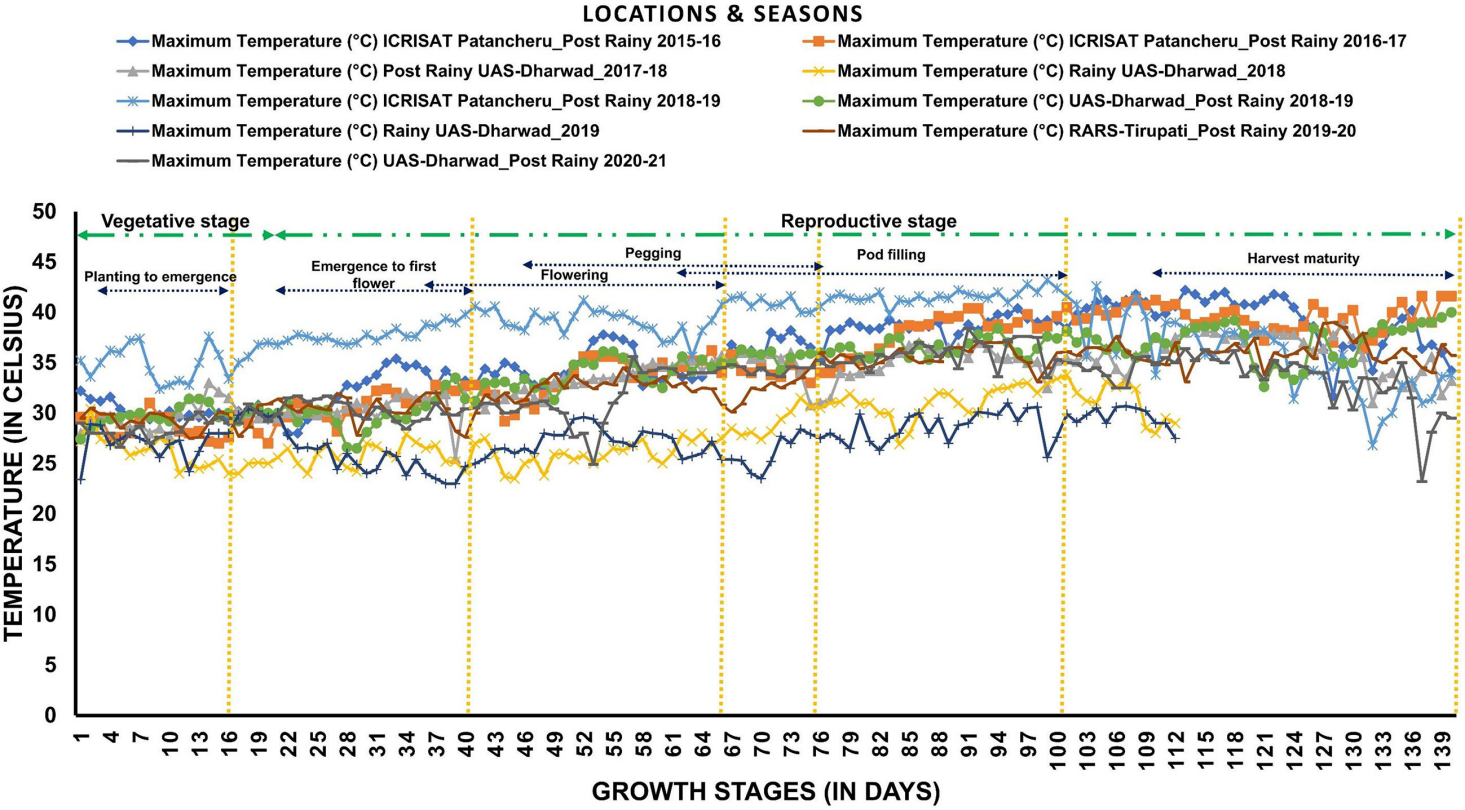
Temperature variability across locations and seasons: The graph shows daily maximum temperatures during each growth stage of groundnut during eight seasons at three locations (ICRISAT-Patancheru, UAS-Dharwad, and RARS-Tirupati).

The RIL population along with parents was evaluated in eight seasons for 6 years (**Table 1** and **Figure 1**) at three geographic locations, namely, ICRISAT Patancheru (17.530′N, 78.270′E), University of Agricultural Sciences (UAS) Dharwad (15.4889′N, 74.9813′E), and Regional Agricultural Research Station (RARS) Tirupati (13.6250′N, 79.3728′E). Seven experimental trials were carried out during the late post-rainy season (second and last weeks of January) to expose the RILs to heat stress during the reproductive stages, and two normal trials were carried out during the rainy season (second week of July). The temperatures were recorded daily during the entire cropping season for 6 years (**Figure 2**). Plants were sown at a spacing of 30 × 10 cm in the field in two replications. At each location, all experimental procedures were executed according to the suggested agronomical practices. The temperature and duration of stress were higher during the reproductive stage, making the study suitable for evaluating RILs for heat stress tolerance during post-rainy trials. Soil moisture and temperatures were measured using the Field Scout TDR 350 Soil Moisture Meter.

**Table 1:** Summary of phenotyping data generated for 31 agronomic, physiological, and phenological traits on RIL population JL 24 × 55-437 across three locations and eight seasons.

Traits	PR 2015–2016 (S1)	PR 2016–2017 (S2)	PR 2017–2018 (S3)	R 2018 (S4)	PR 2018–2019 (S5)	R 2019 (S6)	PR 2019–2020 (S7)	PR 2020–2021 (S8)
Agronomic traits								
Pod weight (kg/ha)	L1	L1	–	–	L1	–	–	–
Pod weight (g plant^−1^)	–	–	L2	L2	L2	L2		L2
Shelling percentage (%)	L1	L1	L2	L2	L1 and L2	L2	L3	L2
100-seed weight (g)	L1	L1	L2	L2	L1 and L2	L2	–	L2
Pod yield per plant (g plant^−1^)	–	–	L2	L2	L2	L2	L3	–
Seed weight (g plant^−1^)	–	–	L2	L2	L2	L2	L3	L2
Shell weight (g plant^−1^)	–	–	L2	L2	L2	L2	–	L2
Biological yield (g plant^−1^)	–	–	L2	L2	L2	L2	–	–
Sound mature kernel percentage (%)	–	–	–	–	L2	L2	–	L2
Harvesting index (%)	–	–	L2	L2	L2	L2	–	–
Physiological traits								
Plant height (cm)	–	–	L2	L2	L2	L2	–	–
Number of primary branches	–	–	L2	L2	L2	L2	–	–
Number of secondary branches	–	–	L2	L2	L2	L2	–	–
Heat use efficiency	–	–	L2	L2	L2	L2	–	–
SPAD chlorophyll meter reading (45 and 70 DAS)	–	–	L2	L2	L2	L2	L3	L2
Canopy temperature (45 and 70 DAS)			L2	L2	L2	L2	L3	L2
Specific leaf weight (g/cm^2^) (70 DAS)	–	–	–	–	L2	L2	–	–
Specific leaf area (cm^2^/g) (45 and 70 DAS)	–	–	–		L2	L2	L3	L2
Membrane injury index (%)	–	–	–	–	–	L2	–	–
Relative water content (%)	–	–	–	–	–	–	L3	–
Leaf area (45 and 70 DAS)	–	–	–	–	–	–	–	L2
Leaf dry weight (45 and 70 DAS)	–	–	L2	L2	L2	L2	–	L2
Stem dry weight (g plant^−1^)	–	–	L2	L2	L2	L2	–	–
Total haulm weight (g plant^−1^)	–	–	L2	L2	L2	L2	–	–
Phenological traits								
Flower initiation	–	–	L2	L2	L2	L2		L2
Days to 50% flowering	–	–	L2	L2	L2	L2		L2
Pod-to-flower ratio	–	–	L2	L2	L2	L2		–
Growing degree of days for flower initiation	**-**	**-**	L2	L2	L2	L2		–
Growing degree of days for Days to 50% flowering	**-**	**-**	L2	L2	L2	L2		–
Phenothermal index for flower initiation	**-**	**-**	L2	L2	L2	L2		–
Phenothermal index for 50% flowering	**-**	**-**	L2	L2	L2	L2		**-**

PR, post-rainy; R, rainy; L1, ICRISAT-Patancheru; L2, UAS-Dharwad; L3, RARS-Tirupati; -, not available; RIL, recombinant inbred line; DAS, days after sowing.

#### 2.2.1 Phenotyping for agronomic traits

The mapping population was phenotyped for agronomic traits like pod weight (PW), hundred seed weight (HSW), shell weight (ShW), biological yield (BY), sound mature kernel percentage (SMKP), shelling percentage (SP), harvest index (HI), and pod yield per plant (PYPP). The traits BY, PYPP, PW, and ShW were calculated by selecting 10 plants at random after reaching maturity and averaging their yields. HI is computed as the ratio of pod weight to biomass. Shelling percentage is measured by shelling the known weight of pods and weighing the kernels obtained after shelling. Mature, sound, and healthy kernels were selected, weighed, and recorded as sound mature kernel percentages. HSW is expressed in grams (g), whereas SP and HI were expressed in percent (%).

#### 2.2.2 Phenotyping for physiological traits

Observations were recorded for physiological traits like plant height (PH), number of primary branches per plant (NPB), number of secondary branches per plant (NSB), leaf dry weight (LDW), stem dry weight (SDW), haulm weight (HW), SPAD chlorophyll meter reading (SCMR), leaf area (LA), specific leaf area (SLA), canopy temperature (CT) in different growth stages like 45 and 70 DAS, specific leaf weight (SLW), relative water content (RWC) at 75 DAS, heat use efficiency (HUE), and membrane injury index (%) (MIIP). Ten plants were selected at random, and the average of the 10 plants was used to record PH, NPB, NSB, and LA. The LDW, SDW, and HW were also recorded and expressed in grams (g). The leaf area was measured with an LI-3100 leaf area meter (Licor Biosciences, Lincoln, NE, USA), and the leaf samples were oven-dried for at least 48 h at 80°C to record the dry weight of the leaf. For the third completely expanded leaf from the top of the main stem, SCMR was measured using a Minolta SPAD 502 (Tokyo, Japan). SLA (cm^2^/g) is the ratio of leaf area to leaf dry weight, whereas SLW (g/cm^2^) is the ratio of leaf dry weight to leaf area. To determine RWC, leaf samples were collected, their fresh weight was recorded, they were soaked for 6 h to record turgid weight, and the leaf samples were oven-dried for at least 48 h at 80°C to record dry weight. RWC was calculated using the formula RWC (%) = [(FW– DW)/(TW– DW)] * 100, where FW stands for fresh weight, DW for dry weight, and TW for turgid weight, as defined by (Barrs and Weartherley, 1962). Canopy temperature (°C) is measured remotely by a Heat Spy infrared thermometer (Palmer Wahl Instrumentation Group, Asheville, NC USA). HUE was given by Rajput (1980) and calculated as the ratio of seed or biomass yield to GDD (day). To estimate membrane injury index (MII), electrical conductivity (EL) was measured before (EC0) and after (ECf) the incubation period whereupon EL starts to stabilize and, ultimately after autoclaving (ECt), expressed as


MII={[(ECf-EC0)-(ECt-EC0)]/[1−(ECt-EC0)]}×100

#### 2.2.3 Phenotyping for phenological traits

Meanwhile, RILs were observed in the field to determine their phenological traits like days to 50% flowering (DFF) and flower initiation (FI), which were calculated from the sowing date and expressed in days. The thermal indices, growing degree days (GDD), and pheno-thermal index (PTI) were calculated by using phenological data and weather data: GDD = Ʃ(Tmax + Tmin)/2) − Tbase, where PTI is the ratio of GDD (day) to the number of days taken between the two phenophases; GDD for flower initiation (GDDFI) and days to 50% flowering (GDDFF) were calculated by the Monteith (1984) formula and expressed in degree Celsius (°C; Tbase, 10°C). GDDPTI for flower initiation (PTIFI) and 50% flowering (PTIFF) were calculated using the Major et al. (1975) formula.

### 2.3 Phenotyping data statistical analysis

Basic statistics of phenotyping data among the 248 RIL individuals for various agronomic, phenological, and physiological traits were performed using the STA extension in QTL IciMapping v4.2 (Wang et al., 2014). The variation for traits was visualized *via* a boxplot using R v3.5.

### 2.4 DNA isolation and sequencing

For each line, a 100-mg tender leaf sample from a 25–30-day-old plant was used for DNA extraction using the Nucleospin Plant II kit (Macherey-Nagel, Düren, Germany; https://guest.link/UM6) as reported in Pandey et al. (2020b). The DNA quality was checked on 0.8% agarose gel, and the DNA for each sample was quantified on Thermo Scientific Nanodrop 2000 Spectrophotometer. GBS was performed according to the protocol reported by Elshire et al. (2011). For GBS, 10 ng of DNA from each line was digested using the restriction endonuclease *Ape*KI. This enzyme recognizes the site G/CWCG, followed by the ligation of barcode adapters to digested products. An equal proportion of adapter-ligated fragments was used for library construction. These libraries were filtered by amplification to remove additional adapters and sequenced on HiSeq 2500 (Illumina Inc., San Diego, CA, USA).

### 2.5 SNP calling and filtering

TASSEL v4.0 was used for SNP discovery from the FASTAQ files of raw sequence reads from the RILs and parents (Bradbury et al., 2007). For SNP calling, the cultivated groundnut *A. hypogaea* cv. Tifrunner.gnm2.J5K5 genome was used as a reference (Bertioli et al., 2019). With the use of the in-house script, the sequencing reads were checked for precisely matched barcodes with the expected four-base remnant of the enzyme cut site. The barcode-containing readings were sorted, de-multiplexed based on the barcode sequence, and trimmed to the initial 64 bases beginning at the enzyme cut site. Furthermore, reads with “N” in the first 64 bases were discarded. With the use of the Burrows–Wheeler Alignment Tool (Li and Durbin, 2009), the remaining high-quality reads (called tags) were aligned to the reference genome of cultivated groundnuts. Subsequently, the alignment file was run through the GBS analysis pipeline to perform SNP calling and genotyping. To avoid false positives, individuals with less than 80 MB of data were not preferred for further analysis. SNPs with contrasting alleles in parental genotypes and <50% missing data with a minor allele frequency (MAF) of ≥0.3 were retained for analysis. With the use of the Beagle version 5.2 algorithm, further imputation of missing data was carried out in RILs (Browning et al., 2018). In addition, filtering was performed to determine the percentage of heterozygosity and polymorphic SNPs between the parents to perform the genetic mapping and QTL analysis.

### 2.6 Construction of genetic linkage map and QTL analysis

The chi-square (χ^2^) values were calculated for each SNP marker in JoinMap v4.0 to determine the goodness of fit to the expected 1:1 segregation ratio for each SNP marker. Highly distorted, monomorphic markers with more than 50% missing calls were filtered out during quality analysis. The linkage map was constructed using JoinMap v4.0 (Van Ooijen, 2006). A regression mapping algorithm was used for grouping and ordering markers. Recombination frequency was converted into map distance (cM) using Kosambi’s mapping function in JoinMap v4.0. A limit of detection (LOD) score threshold of 3.0 was used to construct the linkage group (LG). In this way, the markers were ordered in 20 LGs representing 20 chromosomes of the cultivated groundnut genome. The genetic map information, genotypic data, and phenotypic data were used for composite interval mapping (CIM) implemented in QTL Cartographer v2.5. (Wang et al., 2005). The presence of a significant QTL was decided based on the LOD threshold value of ≥2.5. The QTLs with LOD ≥ 2.5 and phenotypic variation explained (PVE) >10% were considered major main-effect QTLs. With the use of the R software’s LinkageMapView package, the linkage map figure was visualized (Ouellette et al., 2018). Circa software (http://omgenomics.com/circa/) was used to represent the collinearity of the genetic map with reference genomes and to show the major main effect and epistatic QTLs. The effect of alleles on a phenotype of a major main-effect QTL-linked marker was evaluated, and the statistical significance was determined by a Kruskal–Wallis test (Kruskal and Wallis, 1952), using R v3.5.

### 2.7 Identification of epistatic (Q × Q) QTLs

Epistatic QTLs were identified using the Inclusive Composite Interval Mapping for Epistatic (ICIM-EPI) algorithm implemented in ICIM v. 4.1.0.0 with a step of 1.0 cM and 0.001 probability. The LOD threshold was 3.0 as the minimum significance level for defining epistatic QTLs (Wang et al., 2014).

### 2.8 Identification of candidate genes from QTL clusters

For candidate gene discovery, a marker closer to the identified QTL peak was selected, and the genomic region of 2 Mb upstream and downstream was examined. The tissue-specific expression of identified candidate genes was investigated using *A. hypogaea* gene expression atlas (AhGEA) of the *fastigiata* sub-species (BioProject ID: PRJNA484860) (Sinha et al., 2020).

## 3 Results

### 3.1 Phenotypic performance of RILs

**Figure 3:**
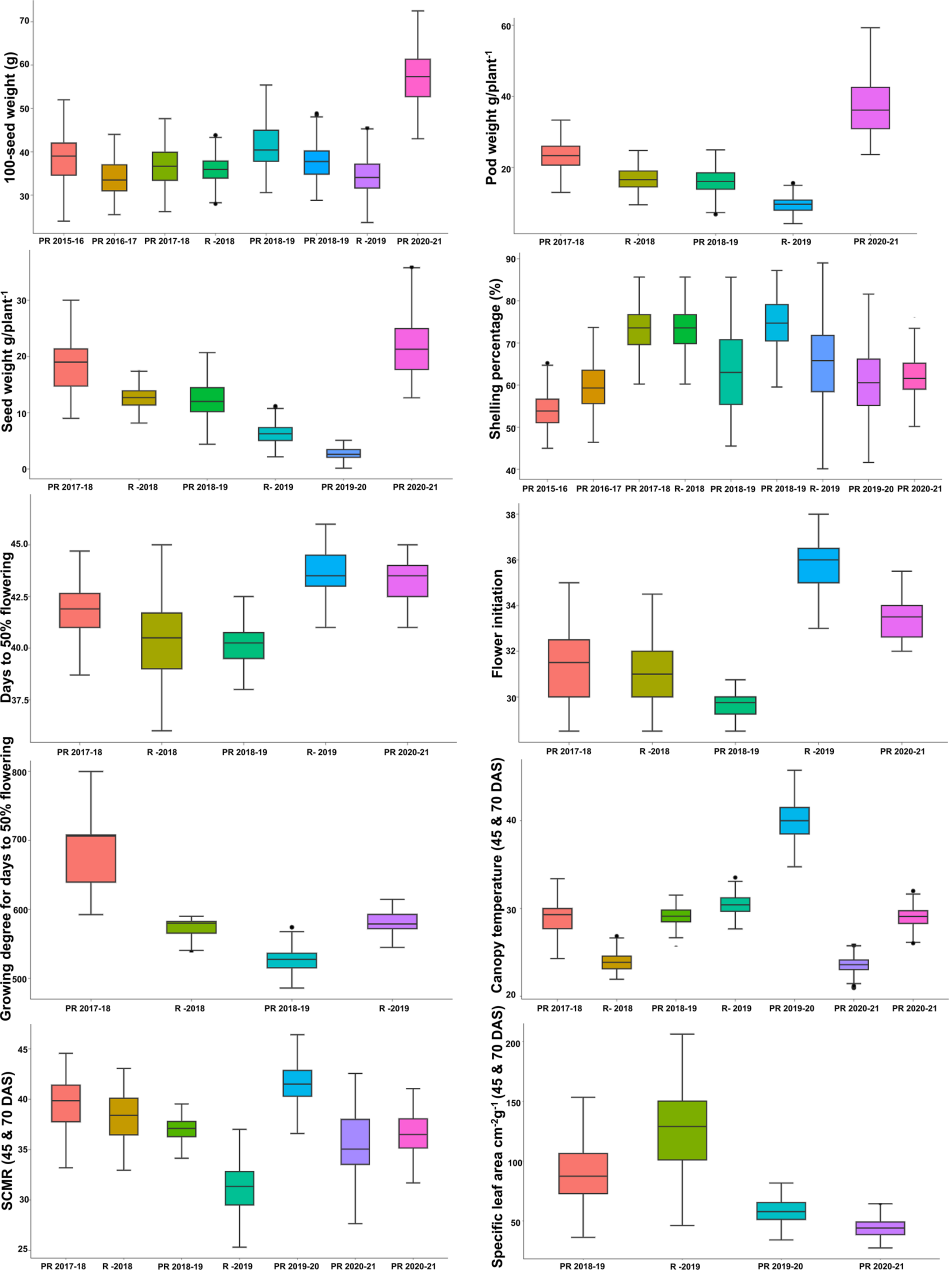
Phenotypic variation in RIL population for different heat tolerance-related traits: boxplot showing variation for agronomic (100-seed weight (g); pod weight (g plant^−1^); seed weight (g plant^−1^), shelling percentage (%), and phenological (days to 50% flowering; flower initiation; growing degree of days for days to 50% flowering) and physiological traits (canopy temperature (45 and 70 DAS); SPAD chlorophyll meter reading (45 and 70 DAS); and specific leaf area cm^2^/g (45 and70 DAS) for the mean value of the RIL population grown over 6 years at three locations. The solid horizontal line represents the median value, the box represents the second and third quartiles, the whiskers represent the 1.5 ± interquartile range, and the dots represent the outlier. RIL, recombinant inbred line; DAS, days after sowing.

The wide range of environmental conditions at three locations over 6 years represented different weather conditions. Phenotypic evaluation of the mapping population showed a high level of genetic variability for agronomic, phenological, and physiological traits in both the parents and the derived RILs. We observed transgressive segregation for some traits among the parents (**Figure 3**). The phenotypic data exhibited normal distribution and represented the quantitative nature of the traits (**Supplementary Figure 1** and **Supplementary Table 1**).

### 3.2 Construction of genetic linkage map

**Figure 4:**
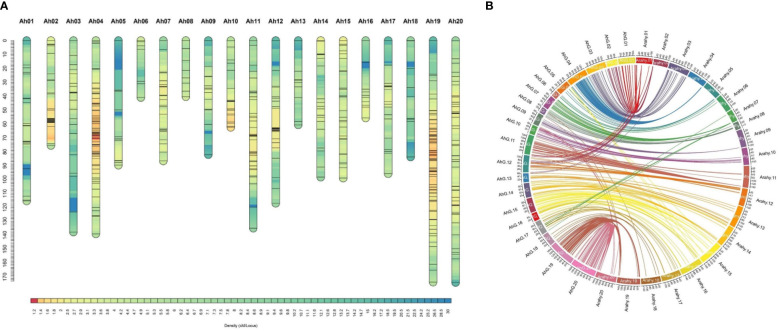
Map density and physical collinearity in genetic map of RIL population: a genetic map of JL 24 × 55-437 RIL population. **(A)** Map charts for 20 linkage groups of groundnut. The color gradient on the bars indicates map density (cM/locus) of each linkage group. **(B)** Circos plot illustrates the collinearity of genetic map with groundnut physical map of reference genome Tifrunner, where the 20 genetic maps (AhG01 to AhG20) are represented by prefix AhG (*Arachis hypogaea* genetic map) and 20 physical maps (Arahy01 to Arahy20) are represented by prefix Arahy (*A. hypogaea* physical map). RIL, recombinant inbred line.

A total of 638.82 million reads (64.5 GB) were generated for 248 RILs and two parental genotypes. The reads from individual lines ranged from 0.80 to 5.77 million reads across the RILs (**Supplementary Table 2**). On average, 2.42 million reads (0.23 GB) of data were generated for each sample. The sequence reads were mapped to the tetraploid reference genome of the cultivated tetraploid groundnut genome represented as arahy.Tifrunner.gnm2.J5K5 (Version 2 from Peanut Base, www.peanutbase.org), and aligned, cleaned GBS reads were used in the pipeline for SNP calling. A total of 34,814 raw SNPs were identified. For instance, 1,127 (Ah08) to 2,952 (Ah16) SNPs were distributed across the chromosomes. Further, SNPs were filtered on percent heterozygosity, MAF, and missing data information. Finally, a total of 677 high-quality polymorphic SNPs between parents were employed to generate a linkage map. Given the stringent selection criteria employed in the present analysis, the number of SNPs was drastically reduced from several thousand to a few hundred. With the use of Joinmap v5.0. χ^2^ test was applied to each SNP to determine the expected Mendelian segregation ratio. The SNPs exhibiting distorted segregation ratios were not used in genetic map construction. A total of 478 high-quality SNP markers were mapped on 20 chromosomes of cultivated tetraploid groundnut. The genetic map spanned a map distance of 1,961.39 cM with an average marker interval of 4.1 cM (**Table 2** and **Figure 4A**). The mapped loci among chromosomes varied from 7 (Ah06) to 68 (Ah19), the map length ranged from 40.3 cM (Ah08) to 173.9 cM (Ah20), and the average marker distance ranged from 2.2 cM (Ah04) to 9.2 cM (Ah18). The maximum inter-marker distance was 35.69 cM (Ah05). The lowest and highest marker densities were recorded for Ah18 (0.11 SNPs/cM) and Ah04 (0.44 SNPs/cM), respectively. Collinearity analysis was performed to evaluate the quality of genetic maps by comparing the genetic (cM) and physical (bp) locations of the SNP loci. This revealed a degree of deviation and translocations between homologous chromosomes (**Figure 4B**).

**Table 2:** Summary of genetic map constructed using single-nucleotide polymorphism (SNP) markers.

Chromosome	Total mapped loci	Map distance (cM)	Average distance (cM)	Max inter-marker distance (cM)	Map density (cM/locus)
Ah01	19	115.30	6.07	19.68	0.16
Ah02	28	75.43	2.69	14.69	0.37
Ah03	20	137.65	6.88	25.69	0.15
Ah04	61	138.99	2.28	18.41	0.44
Ah05	12	89.57	7.46	35.69	0.13
Ah06	7	40.93	5.85	22.94	0.17
Ah07	24	86.50	3.60	16.10	0.28
Ah08	8	40.33	5.04	8.52	0.20
Ah09	13	81.97	6.31	21.81	0.16
Ah10	20	62.38	3.12	13.03	0.32
Ah11	35	134.95	3.86	15.11	0.26
Ah12	26	117.02	4.50	21.49	0.22
Ah13	9	60.60	6.73	22.02	0.15
Ah14	20	98.23	4.91	23.73	0.20
Ah15	26	98.89	3.80	11.89	0.26
Ah16	10	55.62	5.56	19.53	0.18
Ah17	17	95.65	5.63	17.73	0.18
Ah18	9	83.69	9.30	25.58	0.11
Ah19	68	173.72	2.55	24.13	0.39
Ah20	46	173.99	3.78	16.94	0.26
**Total**	**478**	**1,961.39**	**4.10**		**4.60**

### 3.3 QTLs for heat stress tolerance traits

**Figure 5:**
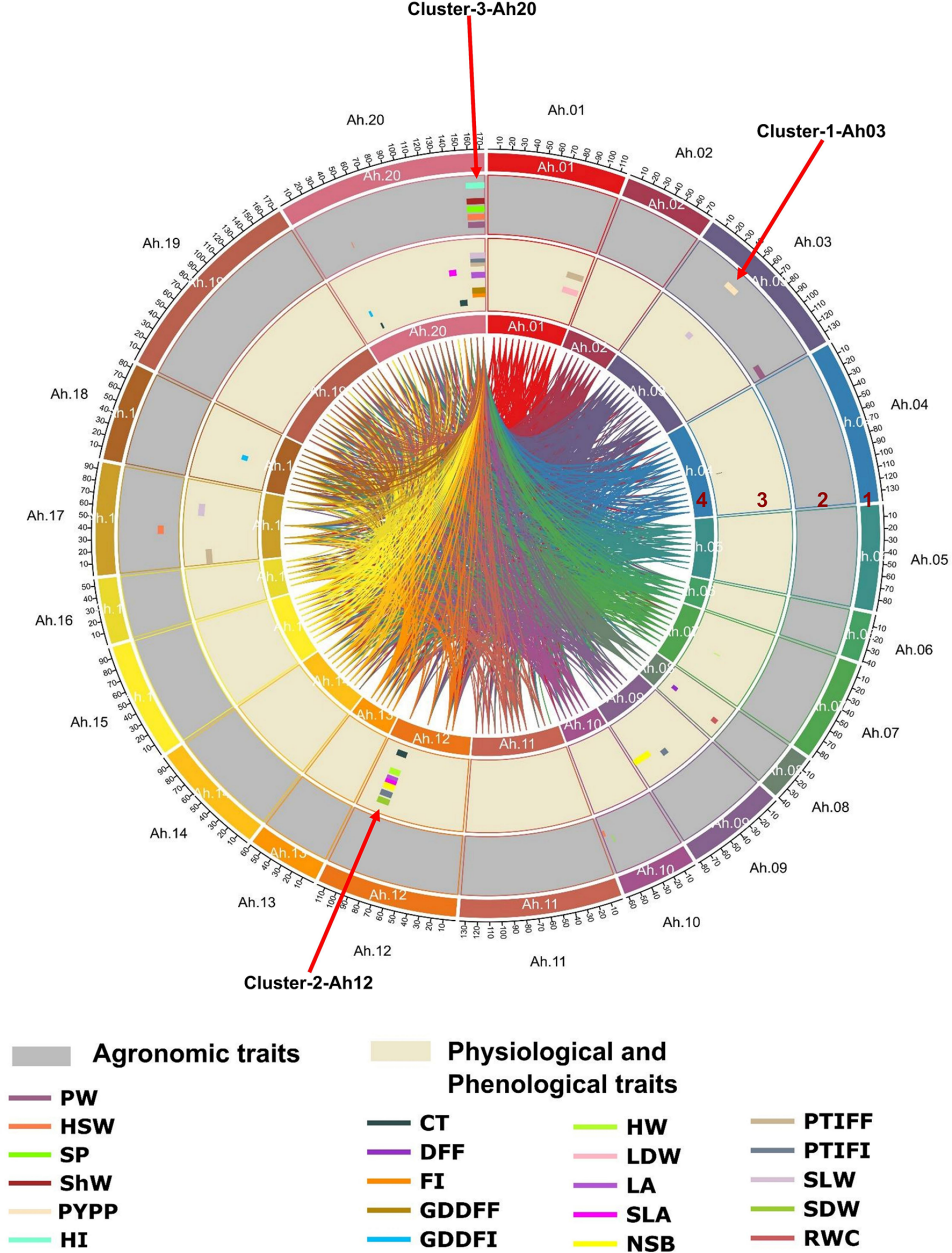
Genome-wide main effect and epistatic QTLs for heat tolerance-related traits in RIL population: Circos plot illustrating major main effect and epistatic (QTL × QTL) QTLs: the tracks from outside to inside indicate 1) 20 chromosomes of tetraploid genome *Arachis hypogaea* L., 2) major main-effect QTLs for agronomic traits (PW, pod weight; HSW, hundred seed weight; SP, shelling percentage; ShW, shell weight; PYPP, pod yield per plant; HI, harvest index), 3) major main-effect QTLs for physiological and phenological traits (CT, canopy temperature; DFF, days to 50% flowering; FI, flower initiation; GDDFF, growing degree days to 50% flowering; HW, haulm weight; LDW, leaf dry weight; LA, leaf area; SLA, specific leaf area; NSB, number of secondary branches; PTIFF, phenothermal index to 50% flowering; SLW, specific leaf weight; SDW, stem dry weight; RWC, relative water content), and 4) epistatic QTLs for agronomic, physiological, and phenological traits shown by the innermost links. QTLs, quantitative trait loci; RIL, recombinant inbred line.

A total of 118 QTLs were identified for 21 traits phenotyped for eight seasons at three locations (ICRISAT-Patancheru, UAS-Dharwad, and RARS, Tirupati) (**Supplementary Table 3**). Of 118 QTLs, 45 QTLs were identified as major main-effect QTLs explaining ≥10% PVE, and 73 QTLs were identified as minor QTLs explaining <10% PVE (**Table 3** and **Figure 5**). Among 45 major main-effect QTLs, 13 QTLs were identified for agronomic traits, 12 QTLs for phenological traits, and 20 QTLs for physiological traits. For each QTL, a positive value for the additive variance of a particular QTL indicated the allele effect from the donor parent (55-437), while negative additive variance indicates the allele effect from the recipient parent (JL 24).

**Table 3:** Major main-effect QTLs for agronomic, physiological, and phenological traits under heat stress.

Trait	Season	Location	QTL name	Chromosome	Position (cM)	Left marker	Right marker	LOD	PVE%	Additive effect
Agronomic traits										
Pod weight (g plant^−1^)	Post-rainy 2017–2018 (S3)	L2	*qPWAh03*	Ah03	134	Ah03_143434398	Ah03_142323610	4.42	10.4	−1.26
	Rainy 2018 (S4)	L2	*qPWAh20*	Ah20	167	Ah20_143291429	Ah20_134973352	4.30	22.4	−2.48
100-seed weight (g)	Post-rainy 2020–2021 (S8)	L2	*qHSWAh10*	Ah10	60	Ah20_46617908	Ah10_96266377	2.60	12.0	−3.24
	Post-rainy 2018–2019 (S5)	L1	*qHSWAh17*	Ah17	38	Ah17_26419006	Ah07_78345818	3.37	15.0	−2.46
	Post-rainy 2018–2019 (S5)	L2	*qHSWAh20.1*	Ah20	40	Ah20_143886635	Ah20_143683219	3.11	10.7	−1.43
	Rainy 2018 (S4)	L2	*qHSWAh20.2*	Ah20	163	Ah20_143291429	Ah20_134973352	6.53	41.6	−2.63
Shelling percentage	Post-rainy 2019–2020 (S7)	L3	*qSPAh10*	Ah10	59	Ah20_46617890	Ah20_46617908	10.61	19.3	−16.24
	Post-rainy 2020–2021 (S8)	L2	*qSPAh20*	Ah20	163	Ah20_143291429	Ah20_134973352	7.17	44.1	−8.53
Shell weight (g plant^−1^)	Rainy 2018 (S4)	L2	*qShWAh20.1*	Ah20	162	Ah20_143291429	Ah20_134973352	3.62	34.7	−1.04
	Post-rainy 2020–2021 (S8)	L2	*qShWAh20.2*	Ah20	162	Ah20_143291429	Ah20_134973352	9.29	49.6	−5.75
Pod yield per plant (g plant^−1^)	Post-rainy 2017–2018 (S3)	L2	*qPYPPAh03.1*	Ah03	48	Ah03_39482436	Ah03_11465956	3.30	11.1	1.50
	Post-rainy 2019–2020 (S7)	L3	*qPYPPAh03.2*	Ah03	51	Ah03_11465956	Ah03_31688785	3.05	11.9	0.82
Harvest index (%)	Post-rainy 2017–2018 (S3)	L2	*qHIAh20*	Ah20	162	Ah20_143291429	Ah20_134973352	7.32	38.2	−0.07
Phenological traits										
Days to 50% flowering	Post-rainy 2017–2018 (S3)	L2	*qDFFAh08*	Ah08	24	Ah08_26119436	Ah18_97934092	3.67	20.7	4.25
Flower initiation	Post-rainy 2017–2018 (S3)	L2	*qFIAh20*	Ah20	164	Ah20_143291429	Ah20_134973352	3.1	22.1	−1.29
Growing degree of days for days to 50% flowering	Post-rainy 2018–2019 (S5)	L2	*qGDDFFAh20*	Ah20	160	Ah20_143291429	Ah20_134973352	6.96	19.4	−17.1
Growing degree of days for flower initiation	Rainy 2018 (S4)	L2	*qGDDFIAh18.1*	Ah18	37	Ah18_3400041	Ah18_25332643	4.68	17.6	5.88
	Rainy 2018 (S4)	L2	*qGDDFIAh20.1*	Ah20	25	Ah20_143886588	Ah20_126361289	5.55	14.8	5.12
Phenothermal index for 50% flowering	Post-rainy 2017–2018 (S3)	L2	*qPTIFFAh01*	Ah01	107	Ah01_38930824	Ah01_249115	4.63	12.7	0.25
	Post-rainy 2017–2018 (S3)	L2	*qPTIFFAh17*	Ah17	6	Ah07_78394898	Ah17_3022700	2.62	10.3	0.2
	Rainy 2018 (S4)	L2	*qPTIFFAh20*	Ah20	159	Ah20_143291429	Ah20_134973352	5.75	26.2	0.11
Phenothermal index for flower initiation	Post-rainy 2017–2018 (S3)	L2	*qPTIFIAh09*	Ah09	44	Ah09_117710533	Ah09_117711142	6.36	14.2	0.19
	Post-rainy 2017–2018 (S3)	L2	*qPTIFIAh10*	Ah10	62	Ah20_46617876	Ah10_96266396	3.77	15.7	0.24
	Post-rainy 2017–2018 (S3)	L2	*qPTIFIAh12.2*	Ah12	86	Ah02_1308170	Ah12_35005363	7.83	22.6	0.7
	Rainy 2018 (S4)	L2	*qPTIFIAh20*	Ah20	159	Ah20_143291429	Ah20_134973352	4.38	29.6	0.11
Physiological traits										
Canopy temperature (°C)	Post-rainy 2020–2021 (S8)	L2	*qCT70Ah04*	Ah04	81	Ah04_123484568	Ah04_125844637	4.433	11	0.4272
	Post-rainy 2017–2018 (S3)	L2	*qCT70Ah12*	Ah12	88	Ah02_1308170	Ah12_35005363	2.854	14	−0.95
	Post-rainy 2020–2021 (S8)	L2	*qCT70Ah20.1*	Ah20	31	Ah20_143683274	Ah20_126357764	2.938	10.2	−0.039
	Post-rainy 2019–2020 (S7)	L3	*qCT70Ah20.2*	Ah20	143	Ah20_143925737	Ah20_135163340	3.147	13	−0.562
Haulm weight (g plant^−1^)	Rainy 2018 (S4)	L2	*qHWAh07*	Ah07	47	Ah07_78345317	Ah07_76081231	3.741	10.1	1.3384
	Rainy 2018 (S4)	L2	*qHWAh12*	Ah12	88	Ah02_1308170	Ah12_35005363	3.439	20.8	2.2012
Leaf dry weight (g plant^−1^)	Rainy 2018 (S4)	L2	*qLDWAh01*	Ah01	108	Ah01_38930824	Ah01_249115	3.716	10.3	0.7401
Leaf area	Post-rainy 2020–2021 (S8)	L2	*qLA45Ah12*	Ah12	86	Ah02_1308170	Ah12_35005363	3.286	13.2	3.5091
	Post-rainy 2020–2021 (S8)	L2	*qLA70Ah12*	Ah12	86	Ah02_1308170	Ah12_35005363	3.147	10.6	3.1318
	Post-rainy 2020–2021 (S8)	L2	*qLA70Ah20.1*	Ah20	169	Ah20_143291429	Ah20_134973352	3.55	10.6	2.5146
Specific leaf area (cm^2^/g)	Post-rainy 2020–2021 (S8)	L2	*qSLAAh12*	Ah12	87	Ah02_1308170	Ah12_35005363	3.402	14.6	4.6915
	Post-rainy 2018–2019 (S5)	L2	*qSLAAh20.1*	Ah20	138	Ah20_143917865	Ah20_143925737	3.137	10.1	8.3013
	Post-rainy 2019–2020 (S7)	L3	*qSLAAh20.2*	Ah20	138	Ah20_143917865	Ah20_143925737	3.777	13.4	11.444
Number of secondary branches	Post-rainy 2017–2018 (S3)	L2	*qNSBAh09*	Ah09	73	Ah09_117711232	Ah19_155135353	3.612	12.2	−0.772
	Rainy 2018 (S4)	L2	*qNSBAh12*	Ah12	86	Ah02_1308170	Ah12_35005363	6.134	28.9	−0.361
Specific leaf weight (g/cm^2^)	Post-rainy 2018–2019 (S5)	L2	*qSLWAh03*	Ah03	51	Ah03_11465956	Ah03_31688785	5.949	38.7	−0.005
	Post-rainy 2018–2019 (S5)	L2	*qSLWAh17*	Ah17	64	Ah07_77404263	Ah17_48527395	8.693	33.4	−0.005
	Post-rainy 2018–2019 (S5)	L2	*qSLWAh20*	Ah20	165	Ah20_143291429	Ah20_134973352	8.132	31.4	−0.005
Stem dry weight (g plant^−1^)	Post-rainy 2018–2019 (S5)	L2	*qSDWAh12*	Ah12	88	Ah02_1308170	Ah12_35005363	6.082	44.6	−2.337
Relative water content	Post-rainy 2019–2020 (S7)	L3	*qRWCAh08*	Ah08	24	Ah08_26119436	Ah18_97934092	2.612	10.7	6.7451

QTLs, quantitative trait loci; LOD, limit of detection; PVE, phenotypic variation explained.

#### 3.3.1 Main-effect QTLs for agronomic traits

A total of 31 main-effect QTLs were identified for six agronomic traits, explaining 4.14% to 49.57% PVE with a LOD score ranging from 2.5 to 10.6, of which 27 were detected in heat stress (HS) conditions and five were identified in non-heat stress (NHS) conditions. Two major main-effect QTLs (*qPWAh03* and *qPWAh20*) were identified for PW on chromosomes Ah03 and Ah20 with the LOD score of 4.4 and 4.3, explaining the phenotypic variance of 10.4 and 22.4% during S3 and S4 season, respectively. For HSW, of the eight QTLs, four QTLs were identified as the major main-effect QTLs. A QTL *qHSWAh10* was identified in season (S8) with a LOD score of 2.6 and a PVE of 12%. The second major main-effect QTL (*qHSWAh17*) on chromosome Ah17 explained 15% PVE with a 3.4 LOD score, while two major main-effect QTLs (*qHSWAh20.1* and *qHSWAh20.2*) were identified over two different seasons (S4 and S5) on the same chromosome, explaining 10.7% and 46.7% PVE. The favorable alleles for PW and HSW major main-effect QTLs were contributed by JL 24. A total of nine QTLs were identified for SP and ShW, of which two major main-effect QTLs were identified for SP (*qSPAh10* and *qSPAh20*) and two major main-effect QTLs (*qShWAh20.1* and *qShWAh20.2*) identified for ShW. Among, these four major main-effect QTLs, three were located on chromosome Ah20 (one for SP and two for ShW), while the second QTL for SP was detected on chromosome Ah10. Furthermore, the PVE for these three QTLs ranged from 19.3% to 49.5%. Eight QTLs were detected for PYPP, of which two were major main effects (*qPYPPAh03.1* and *qPYPPAh03.2*) with PVE of 11.08% and 11.87% and were located on chromosome Ah03. The favorable alleles for PYPP QTLs were contributed by 55-437. A single major main-effect QTL *qHIAh20* was identified for HI on chromosome Ah20, explaining 38.2% PVE and 7.3 LOD with a favorable allele contributed by JL 24.

#### 3.3.2 Main-effect QTLs for phenological traits

For the six phenological traits, a total of 32 main-effect QTLs were identified, which included five QTLs for DFF, four for FI, five for the growing degree of days for GDDFF, six for GDDFI, six for phenothermal index for 50% flowering (PTIFF), and six phenothermal indexes for flower initiation (PTIFI). On chromosomes Ah08 and Ah20, two major main-effect QTLs (*qDFFAh08* and *qFIAh20*) were identified for DFF and FI, explaining 20.73% and 22.13% PVE and 3.7 and 3.1 LOD, respectively. Three major main-effect QTLs (*qGDDFFAh20*, *qGDDFIAh18.1*, and *qGDDFIAh20.1*) were identified on chromosome Ah18 and Ah20 for two traits, GDDFF and GDDFI, explaining 19.41%, 17.62, and 14.77% PVE, respectively. The favorable alleles for DFF and GDDFI were contributed by 55-437, while JL 24 contributed the favorable allele at two major QTLs for FI and GDDFF. Under two seasons (S3 and S4), three major main-effect QTLs (*qPTIFFAh01*, *qPTIFFAh17*, and *qPTIFFAh20*) were identified on chromosomes Ah01, Ah17, and Ah20, with PVE ranging from 10.2% to 26.1%. Moreover, four major main-effect QTLs (*qPTIFIAh09*, *qPTIFIAh10*, *qPTIFIAh12.2*, and *qPTIFIAh20*) associated with PTIFI were identified on chromosomes Ah09, Ah10, Ah12, and Ah20, respectively, with 14.1% to 29.5% PVE. The favorable alleles for PTIFF and PTIFI were contributed by 55-437.

#### 3.3.3 Main-effect QTLs for physiological traits

For the 13 phenological traits, a total of 55 main-effect QTLs were identified, which included 15 main-effect QTLs identified for canopy temperature (°C) at 45 and 70 DAS, of which four were major main-effect QTLs (*qCT70Ah04*, *qCT70Ah12*, *qCT70Ah20.1*, and *qCT70Ah20.2*) and were detected on chromosomes Ah04, Ah12, and Ah20. Among them, the first major main-effect QTL (*qCT70Ah04*) was detected at 70 DAS, explaining 10.9% PVE with a 4.4 LOD score during the S8 season. The second major main-effect QTL, *qCT70Ah12*, identified at 70 DAS, had a PVE of 14.03% with a LOD score of 2.9, during the S3 season. Two major main-effect QTLs (*qCT70Ah20.1* and *qCTAh20.2*) were identified at 70 DAS, over two different seasons (S7 and S8) on the same chromosome (Ah20), explaining 10.21% and 13.01% PVE. The favorable alleles for three QTLs (*qCT70Ah12*, *qCTAh20.1*, and *qCTAh20.2*) were contributed by JL 24, whereas 55-437 contributed the favorable allele for one major QTL (*qCTAh04*).

Twenty-one main-effect QTLs were identified for the four leaf-related traits, including seven QTLs for LDW, five QTLs for LA, six QTLs for SLA, and three QTLs for SLW. Three major main-effect QTLs (*qLA45Ah12*, *qLA70Ah12*, and *qLA70Ah20.1*) for LA at 45 and 70 DAS were identified on chromosomes Ah12 and Ah20, explaining 13.21%, 10.64%, and 10.56% PVE. For SLA, three major main-effect QTLs (*qSLAAh12*, *qSLAAh20.1*, and *qSLAAh20.2*) were identified on chromosomes Ah12 and Ah20. Two QTLs (*qSLAAh20.1* and *qSLAAh20.2*) on chromosome Ah20 were stable across two different seasons and environments. Of them, *qSLAAh20.1* had 10.1% PVE with a LOD of 3.1, and *qSLAAh20.2* had 13.3% PVE with a 3.7 LOD. The third QTL (*qSLAAh12*) detected on chromosome Ah12 had 14.5% PVE and 3.4 LOD. The favorable alleles for LA and SLA were contributed by 55-437. On chromosomes Ah03, Ah17, and Ah20, three major main-effect QTLs (*qSLWAh03*, *qSLWAh17*, and *qSLWAh20*) were identified for SLW, explaining 38.6%, 33.4%, and 31.4% PVE with LOD ranging from 5.9 to 8.6. The favorable alleles for SLW QTLs were contributed by JL 24. A single major main-effect QTL, *qLDWAh01*, for LDW was identified on chromosome Ah01, explaining 10.2% PVE and 3.7 LOD with favorable allele contributed by 55-437.

For the NPB and NSB traits, five main-effect QTLs were identified, of which two were major main-effect QTLs (*qNSBAh09* and *qNSBAh12*) for NSB and located on chromosomes Ah09 and Ah12, explaining 12.2% and 28.9% of the phenotypic variation, respectively. The favorable allele for NSB was contributed by JL 24. Two QTLs were identified for SDW. These include one major main-effect QTL (*qSDWAh12*) on chromosome Ah12, explaining 44.6% PVE and 6.0 LOD with favorable allele contributed by JL 24. One major main-effect QTL (*qRWCAh08*) was identified for RWC on chromosome Ah08, explaining 10.6% PVE with 2.6 LOD. The favorable allele for RWC was contributed by 55-437. For HW, two major main-effect QTLs (*qHWAh07* and *qHWAh12*) were identified on chromosome Ah07 and Ah12, explaining 10.1% and 20.75% of phenotypic variation, respectively. Four main-effect QTLs were detected for HUE on chromosomes A01, A03, A09, and Ah15, with a PVE of 4.1% to 8.6% and a LOD of 2.6 to 4.7. The favorable allele for HW and HUE was contributed by 55-437. For SCMR at 70 DAS, three main-effect QTLs were identified and explained 8.5%–9.1% of the phenotypic variance. Two main-effect QTLs were detected for plant height on chromosome Ah04 and Ah15, explaining 8.2% and 7.9% phenotypic variation, respectively. Additive effect revealed positive allelic contribution from recipient parent JL 24.

### 3.4 Epistatic QTLs identified for agronomic, phenological, and physiological traits

A total of 2,387 epistatic QTLs (E-QTLs) were identified, including 695 E-QTLs for agronomic traits, 742 E-QTLs for physiological traits, and 950 E-QTLs for phenological traits (**Supplementary Table 4**). Several E-QTLs contributed significantly to the phenotypic variations of the assessed traits, ranging from 6.8% to 79.3% for agronomic traits, 6.40% to 62.38% for physiological traits, and 10.01% to 78.51% for phenological traits (**Supplementary Table 5**). Among all detected major E-QTLs, an E-QTL for SP with the highest PVE of 79.3% and 19.54 of LOD showed an interaction between chromosomes Ah04 and Ah10. For DFF, the highest number of E-QTLs (241 E-QTLs) were identified across five seasons (S3, S4, S5, S6, and S8) in environment L2, explaining 11%–78.15% PVE with 3.0–69.8 LOD. For three agronomic traits (BY, SW, and SMKP), one phenological trait (PFR), and one physiological trait (MIIP), no main-effect QTLs were identified. In epistatic QTL analysis, a total of 211 E-QTLs were identified for these agronomic traits, including 10 E-QTLs detected for BY over three seasons (S3–S5) in L2, explaining 7.8%–21.3% PVE with 3.0-3.9 LOD, 60 E-QTLs for seed weight across four seasons (S3–S5 and S8) in L2 with PVE 7.2%–64.7% and LOD of 3.01–8.8, and 141 E-QTLs for SMKP across three seasons (S5, S6, and S8), explaining 10.06%–64.7% PVE and 3.0–22.6 LOD. For phenological traits (PFR), 49 E-QTLs were detected over three seasons (S4–S6) with a LOD score of 3.0–6.9 and a PVE of 21.0%–42.1%. Two major E-QTLs were identified for the physiological trait (MIIP), on chromosomes Ah07 and Ah17, which showed interaction with chromosomes Ah15 and Ah19, explaining 10.8%–17.6% phenotypic variance and 3.01–4.42 LOD.

### 3.5 QTL cluster for agronomic, phenological, and physiological traits

The QTLs identified in this study shared the same confidence intervals and were considered to constitute three major QTL clusters on three chromosomes (Ah03, Ah12, and Ah20). These QTL clusters included more than half of the total major main-effect QTLs (30/45, 66.6%) (**Table 4** and **Supplementary Table 6**). Cluster 1 (Cluster-1-Ah03) on chromosome Ah03 (2.0–137.0 cM) included 13 main-effect QTLs, of which two major main-effect QTLs associated with pod yield, one major main-effect QTL each for pod weight and physiological trait SLW, explained 10.4% to 38.67% phenotypic variation with opposite additive effects (positive and negative). In the case of Cluster-2-Ah12 on chromosome Ah12 (40.0–88.0 cM), 11 main-effect QTLs were identified. Seven major main-effect QTLs for physiological traits and a single major main-effect QTL for phenological traits showed a positive additive effect for CT, HW, LA, SLA, NSB, SDW, and PTIFI. Cluster 3 (Cluster-3-Ah20) on chromosome Ah20 (84.0 to 173.0 cM) harbored 27 main-effect QTLs, of which seven major main-effect QTLs for agronomic traits, five major main-effect QTLs for phenological traits, and six major main-effect QTLs for physiological traits explained 6.08% to 49.6% phenotypic variance with opposite additive effects (positive and negative).

**Table 4:** Distribution of major main-effect QTLs among three clusters for heat tolerance-related traits.

QTL cluster/hotspot genomic regions	Chromosome	Position (cM)	Major main-effect QTLs in cluster region
Cluster-1-Ah03	Ah03	48–134	*qPWAh03* (−), *qPYPPAh03.1* (+), *qPYPPAh03.2* (+), *qSLWAh03* (−)
Cluster-2-Ah12	Ah12	62–88	*qPTIFIAh12.2* (+), *qCT70Ah12* (−), *qLA45Ah12* (+), *qLA70Ah12* (+), *qSLAAh12* (+), *qNSBAh12* (+), *qSDWAh12* (+), *qHWAh12* (+)
Cluster-3-Ah20	Ah20	25–169	*qPWAh20* (−), *qHSWAh20.1* (−), *qHSWAh20.2* (−), *qSPAh20* (−), *qShWAh20.1* (−), *qShWAh20.2* (−), *qHIAh20* (−), *qFIAh20* (−), *qGDDFFAh20* (−), *qGDDFIAh20.1* (−), *qPTIFFAh20* (+), *qPTIFIAh20* (+), *qCT70Ah20.1* (−), *qCT70Ah20.2* (−), *qLA70Ah20.1* (−), *qSLAAh20.1* (+), *qSLAAh20.2* (+), *qSLWAh20* (−)

The “+” and “−” signs in the brackets indicate positive and negative additive effects, respectively, for that particular QTL.

QTLs, quantitative trait loci.

**Figure 6:**
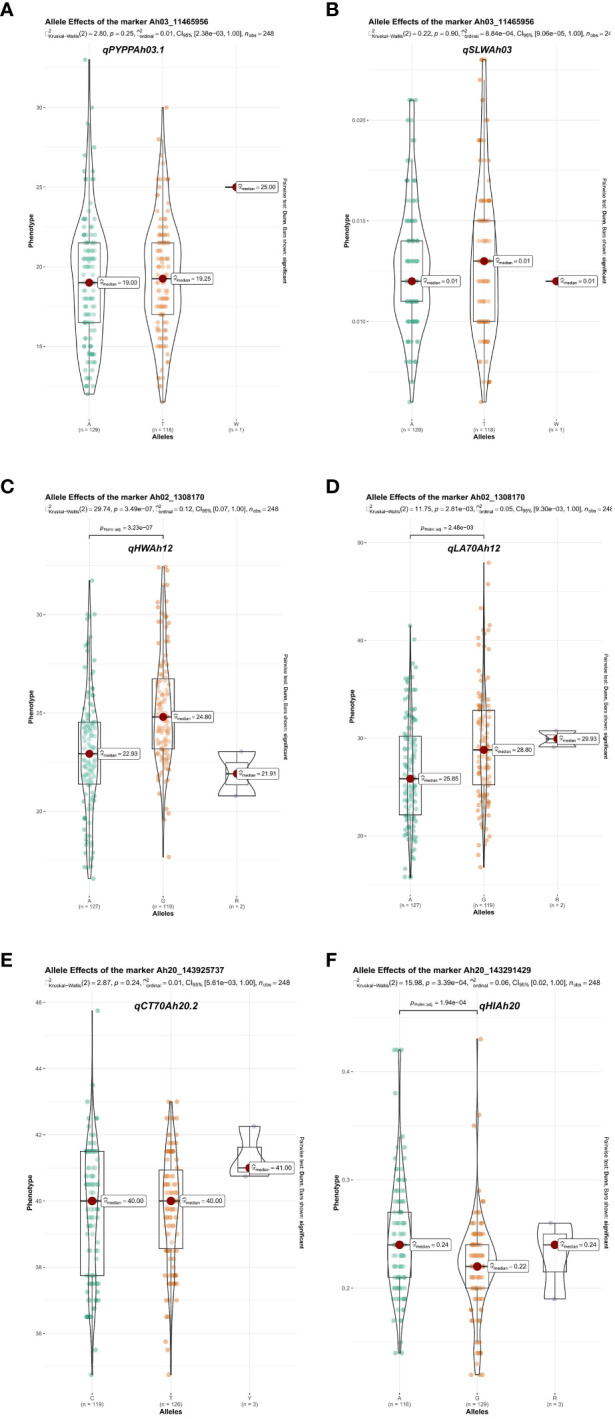
Phenotypic effect of major main-effect QTL favorable alleles for heat tolerance-related traits: phenotype segregation at QTL nearest markers of identified major QTLs underlying QTL clusters. **(A, B)** Phenotype segregation at the *qPYPPAh03.1* and *qSLWAh03* QTLs linked marker (Ah03_11465956) on chromosome Ah03 according to genotype. **(C, D)** Phenotype segregation at the *qHWAh12* and *qLA70Ah12* QTLs linked marker (Ah02_1308170) on chromosome Ah12 according to genotype. **(E, F)** Phenotype segregation at the *qCTAh20.2* and *qHIAh20* QTLs linked markers (Ah20_143925737 and Ah20_143291429) on chromosome Ah20 according to genotype. QTL, quantitative trait locus.

The Kruskal–Wallis test was used to assess the allelic effects of major QTL-linked markers on phenotype for each trait, and their allele segregation as well as p-values (**Figure 6**). For all the traits, the differences among alleles were statistically significant in 24 major main-effect QTLs shown by the pairwise comparison test (**Supplementary Figure 2**). From a breeding standpoint, the differences between alleles were significant, as was the segregation pattern of each marker against the expected ratio in RILs.

### 3.6 Candidate genes in QTL clusters (hotspot genomic) regions

**Figure 7:**
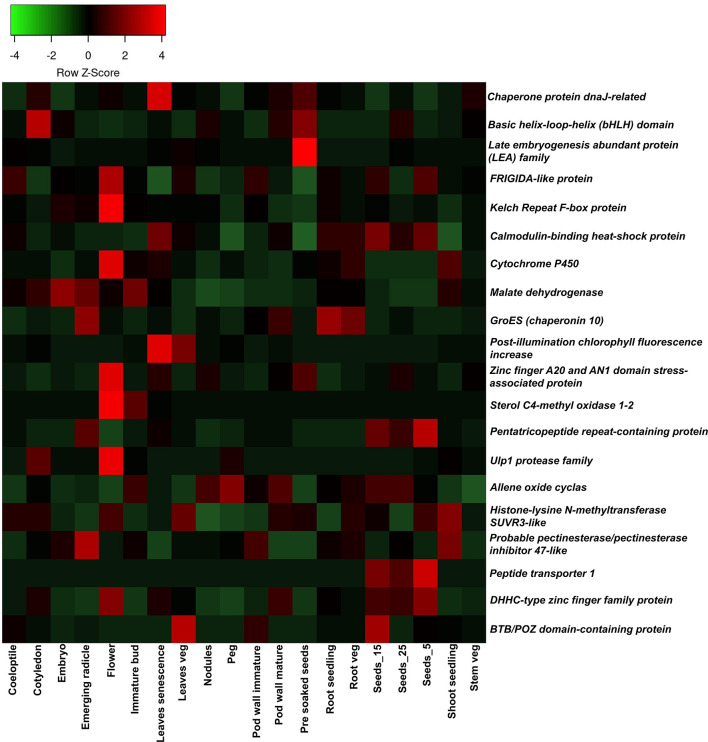
Tissue-specific expression of candidate genes detected underlying three major QTL cluster hotspot genomic regions: the *Arachis hypogaea* gene expression atlas was used to investigate the expression of candidate genes. The expression of 20 candidate genes in 20 organs (coleoptile, cotyledons, embryo, emerging radicle, flower, immature bud, leaves senescence, leaves veg, nodules, peg, pod wall immature, pod wall mature, pre-soaked seeds, root seedling, root veg, Seeds_15, Seeds_25, Seeds_5, shoot seedling, and stem veg) is plotted in the heatmap. QTL, quantitative trait locus.

The genes underlying the marker intervals of three major QTL clusters were retrieved based on the annotated reference genome Tifrunner on peanut base; a total of 1,044 genes were identified in three QTL clusters (**Supplementary Table 7**). A total of 464 genes were identified on chromosome Ah20 in the 17.6 MB region of the overlapping 18 major main-effect QTLs for 14 traits (PW, HSW, SP, ShW, HI, FI, GDDFF, GDDFI, PTIFF, PTIFI, CT, LA, SLA, and SLW). Functional annotation revealed that genes detected in the genomic region are associated with biological processes. Meanwhile, 426 genes were found within overlapping four major main-effect QTLs of three traits (PW, PYPP, and SLW) on chromosome Ah03. For instance, 154 genes were detected in the vicinity of flanking makers of eight major main-effect QTLs on chromosome Ah12. Thirty-six candidate genes (10 on Cluster-1-Ah03, 11 on Cluster-2-Ah12, and 15 on Cluster-3-Ah20) were prioritized based on their role in the regulation of various traits (**Table 5**). With the use of *A. hypogaea* gene expression atlas (AhGEA) of the *fastigiata* sub-species, tissue-specific expression of identified candidate genes was investigated (Sinha et al., 2020). Of the 36 potential candidate genes found in this study, 20 genes were found to be differentially expressed in at least one tissue at key developmental stages in the gene expression atlas across 20 tissues (**Figure 7**).

**Table 5:** Candidate genes identified in the hotspot genomic regions containing major main QTLs for heat tolerance-related traits.

QTL clusters	Gene ID	Start		End	Functional annotation
**Cluster-1-Ah03**	*arahy.SF2S3L*	145303901		145312713	*WD40/YVTN repeat-like-containing domain*
	*arahy.KW7RVG*	145474880		145476745	*Coronatine-insensitive 1*
	*arahy.T0XEU7*	11190158		11194238	*Chaperone protein dnaJ-related*
	*arahy.4C0G14*	11680312		11683789	*Basic helix-loop-helix* (*bHLH*) *domain*
	*arahy.9DDU7S*	11683379		11685988	*Late embryogenesis abundant protein* (*LEA*) *family protein*
	*arahy.625I4R*	38855795		38859896	*Zinc finger-2C CCHC-type*
	arahy.KHTN7F	38994285		38998376	*Auxin efflux carrier family protein*
	*arahy.FP3Q5V*	39161518		39164569	*WRKY family transcription factor*
	*arahy.9E5EUV*	40258049		40259799	*P700 chlorophyll A apoprotein*
	*arahy.GU2JPA*	31445548		31446158	*RING finger protein 38-like*
**Cluster-2-Ah12**	*arahy.9X2P9A*	556893		562588	*Flowering locus protein* (*Phosphatidylethanolamine-binding protein PEBP*)
	*arahy.S5CNIB*	585368		586454	*F-box family protein*
	*arahy.0C3V8Z*	1346201		1357050	*FRIGIDA-like protein*
	*arahy.I7X4PC*	104606		106633	*Kelch Repeat F-box protein*
	*arahy.HQA8FP*	131485		135404	*Calmodulin-binding heat-shock protein*
	*arahy.XKLY96*	349505		351125	*Cytochrome P450*
	*arahy.HLUA2X*	593574		606861	*Malate dehydrogenase*
	*arahy.6U9S6T*	950623		953587	*GroES* (*chaperonin 10*)
	*arahy.92ZGJC*	1036091		1038893	*Post-illumination chlorophyll fluorescence increase*
	*arahy.MVDL02*	1150364		1151392	*HSP20-like chaperone*
	*arahy.7SF3LE*	1324267		1324771	*Zinc finger A20 and AN1 domain stress-associated protein*
**Cluster_3_Ah20**	*arahy.IWD7T4*	126857401		126858061	*Serine/threonine-protein phosphatase*
	*arahy.4A4JE9*	127850959		127852270	*Pentatricopeptide repeat-containing protein*
	*arahy.JXD391*	130800831		130802025	*HARBI1-like*
	*arahy.X568GS*	136167165		136172754	*Ulp1 protease family*,
	*arahy.F4N1WH*	139486363		139488495	*Allene oxide cyclas*
	*arahy.WXUV3N*	140897048		140900442	*Histone-lysine N-methyltransferase SUVR3-like*
	*arahy.6R9DCH*	141413077		141426967	*Cellulose synthase*
	*arahy.430A6X*	141605491		141612713	*Probable pectinesterase/pectinesterase inhibitor 47-like*
	*arahy.E8V3SA*	141627892		141628952	*Flowering locus protein T*
	*arahy.8ZMT0C*	142228210		142232275	*Peptide transporter 1*
	*arahy.J0Y6Y5*	142698324		142701852	*DHHC-type zinc finger family protein*
	*arahy.4572PU*	142773290		142776227	*MYB transcription factor MYB109*
	*arahy.PV785R*	142956262		142959406	*BTB/POZ domain-containing protein*
	*arahy.BITW79*	135644705		135649148	*Sterol C4-methyl oxidase 1-2*
	*arahy.1BXK41*	133573663		133576546	*NAC domain-containing protein 94-like*

QTLs, quantitative trait loci.

## 4 Discussion

In the global context, each degree rise in aerial temperature has a devastating effect on crop productivity, particularly on groundnut, which is mostly grown in SAT regions. Understanding the nature, effects, and underlying processes of heat stress tolerance might assist in reducing production losses. In the past, relatively few research efforts were focused on the nature, effects, and existing diversity in germplasm lines of groundnut. In this investigation, we identified the major main-effect QTLs and candidate genes related to heat stress tolerance. Late sowing, a simple and efficient field screening approach, was adopted to phenotype the RILs for heat stress tolerance. In order to understand the genetic variation for heat stress among genotypes and to detect the genomic regions associated with heat stress tolerance, the late sowing method was earlier adopted in winter crops such as wheat (Sareen et al., 2020), brassica (Branham et al., 2017), and rice (Prasanth et al., 2016). Direct selection based on yield traits tends to lead to a slower response, due to G × E interactions; we also phenotyped RIL for 14 physiological and seven phenological traits, which could be used as an indirect selection criterion to improve heat tolerance in groundnut, similar to other crops.

The RIL population was studied for eight seasons in three different locations. Both parents and RILs showed significant genetic variability for agronomic, physiological, and phenological traits. Similar findings were reported previously in groundnut based on the evaluation of germplasm lines in the HS condition (Singh et al., 2014). In this study, the genotyping of RILs *via* genotyping-by-sequencing facilitated the construction of a genetic map. The tetraploid groundnut (*A. hypogaea*) genome was used as the standard for calling the true representative SNPs (Bertioli et al., 2019). The genetic map comprises 478 SNPs, with a total map length of 1,961.39 cM, an average marker interval of 4.1 cM with average map density of 4.60 SNPs/cM. This is the first study to report QTLs for agronomic, phenological, and physiological traits in groundnuts under HT conditions. A total of 118 main-effect QTLs (31 QTLs for six agronomic traits, 32 QTLs for six phenological traits, and 55 QTLs for physiological traits) were identified; of these, 45 QTLs had major main effects with >10% PVE (13 QTLs for six agronomic traits, 12 QTLs for six phenological traits, and 20 QTL for nine physiological traits). Minor main-effect QTLs have been found in many crops, including groundnut (Pandey et al., 2020b), rice (Roy et al., 2023), and maize (Ribaut et al., 1997), for complex traits that are usually controlled by multiple genes. Indeed, the impact of individual locus may be minor, but the cumulative contribution of loci for such complex traits is often significant.

Interestingly, 30 major main-effect QTLs were found to overlap for agronomic, phenological, and physiological traits on Ah03, Ah12, and Ah20. The QTLs for highly correlated traits, such as FI, GDDFF, GDDFI, PTIFF, and PTIFF, shared a common chromosome Ah20. Similarly, QTLs for PW, HSW, ShW, and SP shared common marker interval QTLs on Ah20 and for CT, LA, SLA, NSB, and SDW on chromosome Ah12. These cluster genomic regions seem to be hotspot genomic regions that could help to improve two or more traits simultaneously. Several studies also investigated QTL clustering for different categories of traits (Deng et al., 2019; Liu et al., 2019). Such studies highlight the importance of simultaneous assessment of various traits and pinpoint the genomic regions associated with the traits of interest. Recently, four QTL clusters for saturated fatty acid content were reported in groundnut (Liu et al., 2019). Clustering of QTLs for several traits may arise when gene frequency changes at closely related loci occur, but the pleiotropic action of genes may also induce it. Additionally, pleiotropy and/or related genes may have contributed to this event (Smith and Haigh, 1974). Three QTL clusters (Cluster-1-Ah03, Cluster-2-Ah12, and Cluster-3-Ah20) harboring major main-effect QTLs on chromosomes Ah03, Ah12, and Ah20 showed opposite additive effects (positive and negative) for agronomic, phenological, and physiological traits, which revealed such traits could be improved synchronously. Developing appropriate selection strategies, identifying unfavorable combinations of QTLs, developing molecular markers, and utilizing genomic information for optimal crosses are effective approaches in such scenarios (Zhang et al., 2020). Two major main-effect QTLs (*qPYPPAh03.1* and *qPYPPAh03.2*) for PYPP with 11.1 and 11.9% PVE were detected on Cluster-1-Ah03. These QTLs were stable and flanked by the same nearest markers on the chromosome across two environments (L2 and L3). This showed their expression was not affected by environmental factors, as found in other crops (Sharma et al., 2017). Similarly, four stable QTLs (*qShWAh20.1*, *qShWAh20.2*, *qSLAAh20.1*, and *qSLAAh20.2*) for two traits (ShW and SLA) were identified on Cluster-3-Ah20. These QTLs were stable and flanked by the same markers on the same chromosome across two seasons (S4 and S8, and S5 and S7).

Ten major main effect stable QTLs, including two on Ah03 (*qPYPPAh03.1* and *qPYPPAh03.2*) and eight on Ah20 (*qHSWAh20.1*, *qHSWAh20.2*, *qShWAh20.1*, *qShWAh20.2*, *qCT70Ah20.1*, *qCT70Ah20.2*, *qSLAAh20.1*, and *qSLAAh20.2*), were found. Other QTLs were strongly influenced by environmental variables and only identified in one season; further confirmation is therefore needed for such QTLs. Several QTLs also showed consistency, while their reported co-localization in prior studies supported their effectiveness. Two QTLs (*qPWAh03* and *qCT70Ah04*) on chromosomes Ah03 and Ah04 identified for PW and CT in this study were consistent with the reported genomic region (Pandey et al., 2020b).

These identified clusters on chromosome-aligned major main-effect QTLs for several traits, which might be targeted for simultaneous trait improvement in groundnut breeding. The findings of several QTL mapping studies indicate that epistasis is a key genetic component underpinning complex traits (Ravi et al., 2011; Varshney et al., 2014). E-QTLs are considered important for both qualitative and quantitative traits, resulting from gene interaction among genomic regions (Yu et al., 1997). Interestingly, in the current study, we identified 2,387 E-QTLs, including 695 E-QTLs for agronomic traits, 742 E-QTLs for physiological traits, and 950 E-QTLs for phenological traits. Previous studies reported the involvement of a large number of E-QTL in morphological and yield-related traits in groundnut (Varshney et al., 2009; Ravi et al., 2011; Pandey et al., 2020b). Furthermore, E-QTLs with major effects linked to drought tolerance were identified and effectively used in genomic-assisted breeding in diverse genetic backgrounds of rice in order to assess productivity under stress conditions (Sandhu et al., 2019; Yadav et al., 2021).

Now that the genome sequences of both the diploid ancestors and the cultivated groundnut are available (Bertioli et al., 2019), identifying candidate genes is not very challenging (Pandey et al., 2020a). Based on the QTL analysis, candidate genes co-localized in the QTL-clusters genomic regions were identified. This study reported several candidate genes and transcription factors in the three major QTL clusters’ genomic regions, responsible for various signaling pathways and acting as key transcriptional regulators in plants. The hotspot genomic region of the QTL cluster (Cluster-1-Ah03) on chromosome Ah03 harboring QTLs for PW, PYPP, and SLW was selected to identify putative candidate genes controlling these traits. Relying on such premises, 10 genes underlying Cluster-1-Ah03 genomic region and some of these genes have been demonstrated to play an important role in seed development in prior research. For instance, a gene encoding *Coronatine-insensitive 1* (*arahy.KW7RVG*) was involved in jasmonic acid signaling and was found to be linked with spikelet fertility and grain weight (Lee et al., 2015). Similarly, the *bHLH* (*basic helix-loop-helix*) transcription factor (*arahy.4C0G14*) and *WD40/YVTN repeat-like-containing domain* (*arahy.SF2S3L*) genes were shown to play a significant role in controlling seed development (Heang and Sassa, 2012; Hu et al., 2018). A *RING finger protein* (*arahy.GU2JPA*) family gene was also linked to levels of active cytokinins, which control plant architecture and grain number (Yan et al., 2022). Furthermore, the *chaperone protein dnaJ-related* (*arahy.T0XEU7*) gene, which acts as a molecular chaperone, performed crucial roles in the genesis and growth of plants, as well as in their responses to heat stress (Fan et al., 2017). *Late embryogenesis abundant* (*LEA*) (*arahy.9DDU7S*) genes encode a large family of proteins found in many plants. They are mostly linked to desiccation mechanisms during the development of plants or responding to abiotic stresses (Zheng et al., 2019). *Zinc finger CCHC-type* (*arahy.625I4R*) genes regulate plant growth, development, and stress adaptation (Han et al., 2021). Furthermore, the *auxin efflux carrier family protein* (*arahy.KHTN7F*) and *WRKY family transcription factor* (*arahy.FP3Q5V*) displayed their roles in plant responses to high temperature (Cheng et al., 2021) and water stress (Zhang et al., 2012).

Similarly, in the second cluster (Cluster-2-Ah12), plausible candidate genes underlying the mapped QTLs were identified. Genes encoding *flowering locus protein T* (*arahy.9X2P9A*), *FRIGIDA-like protein* (*arahy.0C3V8Z*), and *Kelch repeat F-box protein* (*arahy.I7X4PC*) were found to be associated with regulation of flowering time (Vieira et al., 2019; Jin et al., 2021; Shibuya et al., 2021). The *F-box family protein* (*arahy.S5CNIB*) family genes regulate antioxidant competence and accumulation of ROS under stress conditions (An et al., 2019). A *calmodulin-binding heat-shock protein* (*arahy.HQA8FP*) encoding gene plays a key role in activating and expressing the heat-shock transcription factor and genes for improving plant heat tolerance (Zhou et al., 2009). Furthermore, the *post-illumination chlorophyll fluorescence increase gene* (*arahy.92ZGJC*) was associated with an indirect effect on photosynthesis in *Arabidopsis* (Gotoh et al., 2010). Moreover, *malate dehydrogenase* (*arahy.HLUA2X*), *GroES* (*chaperonin 10*) (*arahy.6U9S6T*), and *HSP20-like chaperone* (*arahy.MVDL02*) were underlying in Cluster-2-Ah12 hotspot genomic region. Integrated omics strategies give insight into the function of these genes in drought and heat stress responses. *Malate dehydrogenase* genes are known to play a key role in photorespiration (Tomaz et al., 2010), and *chaperonin 10* is involved in protein folding, regulation of metabolic processes, and abiotic stress responses (Pareek et al., 2021). An *A20/AN1-type zinc finger* (*arahy.7SF3LE*) protein is involved in regulating gibberellins and abscisic acid levels and improves rice’s growth and stress response (Zhang et al., 2016).

Additionally, 15 potential genes underpin Cluster-3-Ah20 hotspot genomic region. The *arahy.IWD7T4* gene was reported to encode for *serine/threonine-protein phosphatases*, which may be the operators of signal transduction cascades and have been identified as an essential factor for cell division and differentiation in *Arabidopsis* (Ühlken et al., 2014). *MYB transcription factors* (arahy.4572PU) are involved in hormone signal transduction, and abiotic stress tolerance (Zhang et al., 2018). *DHHC-type zinc finger family protein* (*arahy.J0Y6Y5*) genes are involved in the regulation of plant architecture and grain yield (Zhou et al., 2017). The gene *arahy.4A4JE9* encodes a *pentatricopeptide repeat-containing protein* that regulates the development of the kernel in maize (Ren et al., 2019). *BTB/POZ domain-containing protein* (*arahy.PV785R*) genes are substrate adaptors for *Cullin3-based E3 ubiquitin ligase*, regulating the heat stress response and preventing the adverse effects of excess *DREB2A* on plant growth (Morimoto et al., 2017). Similarly, the *arahy.JXD391* gene encodes *HARBI1-like*, and its homolog *MdHARBI1* plays a positive role in plant thermos tolerance (Huo et al., 2021). Genes including the *Ulp1 protease* family (*arahy.X568GS*) and *flowering locus protein T* (*arahy.E8V3SA*) have been shown to play a major role in the regulation of flowering in plants (Murtas et al., 2003; Pieper et al., 2021). The *cellulose synthase* family (*arahy.6R9DCH*) gene is involved in cell division and controls plant organ development (Li et al., 2018). *Allene oxide cyclas* (*arahy.F4N1WH*) gene involvement in jasmonic acid homeostasis in cotton has been reported under high-temperature stress (Khan et al., 2023). Moreover, *peptide transporter 1* (*arahy.8ZMT0C*) family sugar transporter genes that regulate seed filling and enhance yield have been reported in maize and other crops (Yang et al., 2022). A gene encoding the *sterol C4-methyl oxidase 1-2 enzyme* (*arahy.BITW79*) was found to be involved in the sterol biosynthetic pathway and to regulate embryogenesis in *Arabidopsis via* auxin and cytokinin homeostasis (Song et al., 2019). A gene *arahy.1BXK41* encodes the *NAC domain-containing protein*, which is involved in starch and storage protein synthesis in rice (Wang et al., 2020).

With the use of the *AhGEA* gene expression atlas, the tissue-specific expression pattern of the aforementioned genes was investigated (Sinha et al., 2020). Consequently, 20 genes were found to be differentially expressed in at least one tissue at key developmental stages and were involved in seed development, plant architecture, grain number, the genesis and growth of plants, desiccation mechanisms, flowering time regulation, and photosynthesis. Higher expression levels of the *DHHC-type zinc finger family protein*, *peptide transporter 1*, and *pentatricopeptide repeat-containing protein* were observed in the seed developmental stages. *Allene oxide cyclas* showed a higher expression pattern at all stages of the seed. *Kelch Repeat F-box protein* and *Ulp1 protease family* were noticed to be highly expressed in the flower tissue. Based on these findings, genes should be targeted for fine mapping and gene cloning in order to determine the genetic link of traits in groundnut. Further investigation of haplotypes for the genes reported here using diverse germplasm sequencing information might lead to genetic improvement of the trait.

## 5 Conclusion

Genome-wide QTL analysis identified 45 major main-effect QTLs for 21 traits. Most importantly, we detected more than half of the major main-effect QTLs in three QTL clusters that explained 10.1%–49.5% of phenotypic variance, which seems to be the hotspot genomic region controlling heat tolerance-related traits. Collectively, such QTL cluster hotspot genomic regions provide a basis for improving several traits simultaneously, but fine mapping of such QTL-rich intervals on particular chromosomes is necessary for their future use in MAS and candidate gene cloning. We can also use QTL cluster information on the linkage disequilibrium (LD) structure to select markers that are in LD with multiple QTLs to improve the efficiency of MAS. Furthermore, important candidate genes encoding chaperone proteins, flowering-related genes, plant architecture, and yield-regulating genes were underlying genomic regions of QTL clusters. The AhGEA expression atlas revealed 20 candidate genes’ expression patterns at key developmental stages, which will be important to understand their precise role. Such candidate genes can help to identify the molecular targets, provide insights into the biological pathways underlying the traits of interest, and help in comprehending the genetic basis of complex traits. Targeting these QTL clusters for future research would aid in understanding heat tolerance mechanisms and functional markers for genomics-assisted breeding for accelerated development of heat-tolerant lines/varieties.

## Data availability statement

The data presented in the study are deposited in NCBI, accession number PRJNA973209.

## Author contributions

Conceptualization (MP), investigation (VS, MP, SSG, RB, PJ, SNN (3rd author), and PL), resources (MP, PJ, SNN (3rd author), RB, and PL), methodology (VS, SSG, SNN (3rd author), RB, PL, SSM (6th author), SSM (7th author), SNN (11th author), ASG, BS, SSM (10th author), SS, TA, MP, and RC), data curation and formal analysis (VS, SSG, and PB), and writing—review and editing (VS, SSG, MP, SNN (3rd author), RB, PL, SS, and RV). All authors contributed to the article and approved the submitted version.
